# Metal-based immunogenic cell death inducers for cancer immunotherapy

**DOI:** 10.1039/d4sc08495k

**Published:** 2025-02-25

**Authors:** Jiao Xia Zou, Meng Rui Chang, Nikita A. Kuznetsov, Jia Xuan Kee, Maria V. Babak, Wee Han Ang

**Affiliations:** a Department of Chemistry, National University of Singapore 4 Science Drive 2 Singapore 117544 Singapore ang.weehan@nus.edu.sg; b Drug Discovery Lab, Department of Chemistry, City University of Hong Kong 83 Tat Chee Avenue Hong Kong SAR 999077 People's Republic of China mbabak@cityu.edu.hk; c NUS Graduate School – Integrative Science and Engineering Programme (ISEP), National University of Singapore 21 Lower Kent Ridge Rd Singapore 119077 Singapore

## Abstract

Immunogenic cell death (ICD) has attracted enormous attention over the past decade due to its unique characteristics in cancer cell death and its role in activating innate and adaptive immune responses against tumours. Many efforts have been dedicated to screening, identifying and discovering ICD inducers, resulting in the validation of several based on metal complexes. In this review, we provide a comprehensive summary of current metal-based ICD inducers, their molecular mechanisms for triggering ICD initiation and subsequent protective antitumour immune responses, along with considerations for validating ICD both *in vitro* and *in vivo*. We also aim to offer insights into the future development of metal complexes with enhanced ICD-inducing properties and their applications in potentiating antitumour immunity.

## Abstract 2

A comprehensive review of metal-based inducers of immunogenic cell death (ICD), their design strategies, molecular mechanisms to trigger ICD, subsequent protective antitumor immune responses, as well as validation approaches.

## Graphical abstract

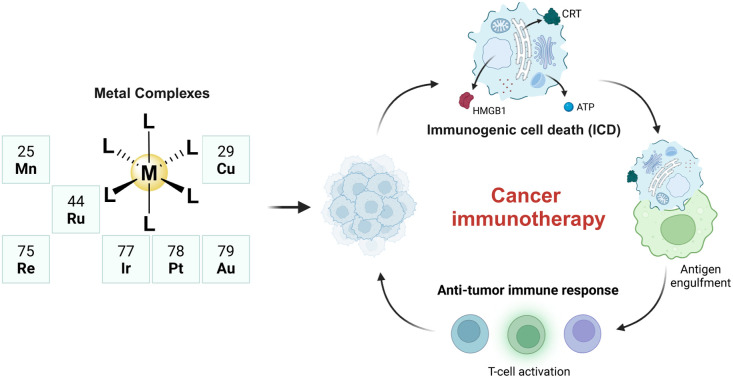

## 1. Introduction

The landscape of clinical cancer treatments has undergone a significant transformation with the advent of immunotherapy, driven by the rise of revolutionary technologies, such as immune checkpoint blockade therapy,^1^ adoptive T-cell therapy,^2–4^ and cancer vaccines.^5^ The concept of harnessing the body's immune system to combat cancerous cells dates back to the 1800s, when physicians Fehleisen and Busch observed tumour regression in cancer patients infected with *Streptococcus pyogenes*-induced erysipelas.^6,7^ In 1891, William Bradley Coley, acknowledged as the Father of Immunotherapy, first injected inactivated bacteria (“Coley's toxins”) to activate the immune system for treating bone cancer, thereby pioneering the field of cancer therapy.^8^

Immunotherapy offers a distinct advantage over conventional cancer treatment modalities, such as surgery, chemotherapy, and radiotherapy, due to its systemic tumour-targeting capability and its potential to confer sustained, long-term immunity against tumours.^9^ However, the success of immunotherapies is heavily linked to the state of an individual's immune system and the immunogenicity of the tumour.^10^ The complexity of the immune response, negative feedback loops, immune evasion checkpoints, cancer heterogeneity, and other factors further complicate the efficacy of immunotherapies.^11–18^ In light of the challenges and limitations of cancer immunotherapy, tremendous efforts have been made to identify key determinants of anticancer immune responses to improve immunotherapy outcomes.^19^ Within the cancer-immunity cycle, a crucial factor in initiating an immune response against cancer involves the recognition of cancer antigens by the immune system.^20^ However, tumours can reduce their immunogenicity through multiple mechanisms, such as upregulating PD-L1, secreting immunosuppressive factors, and establishing an immunosuppressive tumour microenvironment (TME) that is inaccessible to immune cells.^15,21–24^

Enhancing tumour immunogenicity has emerged as a promising strategy to combat tumour-induced immunosuppression. Immunogenic cell death (ICD), defined by the Nomenclature Committee on Cell Death as a form of regulated cell death (RCD) that is sufficient to activate an adaptive immune response in immunocompetent syngeneic hosts,^25,26^ has been at the forefront of this approach. ICD was first recognized in 2005 by Kroemer and coworkers, who found that doxorubicin (DOX)-induced apoptotic tumour cell death is immunogenic. They demonstrated that DOX-treated tumour cells can serve as cancer vaccines to elicit antitumour immune responses mediated by dendritic cells (DCs) and cytotoxic CD8^+^ T-cells in immunocompetent mice.^27^ This study first linked apoptosis inflicted by certain chemotherapeutic agents to ICD. Apoptosis, traditionally considered a physiological form of cell death, was believed to be immunogenically silent or even immunosuppressive for many years.^28–30^ Following the discovery of anthracycline-induced ICD phenomenon, other chemotherapeutic agents, such as mitoxantrone (MTX)^31,32^ and oxaliplatin (OXP),^33^ cardiac glycosides,^34^ as well as some physical anticancer therapies, including γ-irradiation^35^ and photodynamic therapy,^36,37^ were also reported to induce ICD, resulting in antitumour immune response *in vivo*. These studies on ICD open the possibility of fully using ICD as an effective strategy to modulate the innate and adaptive immune systems, with the aim of preventing tumour recurrence and metastasis.

**Fig. 1:**
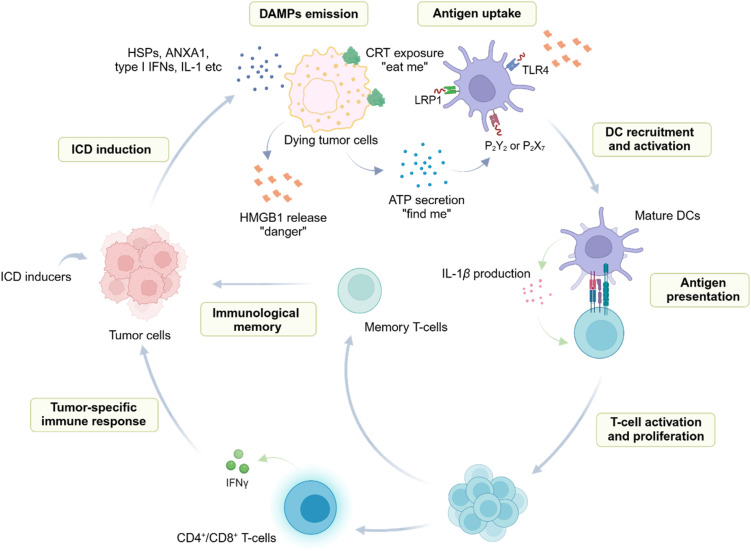
Activation of antitumour immune response following ICD induction. Upon treatment with ICD inducers, tumour cells undergo ICD and emit DAMPs. DAMPs recruit immune cells to the ICD site and interact with their corresponding receptors (*i.e.* CRT-LRP1, HMGB1-TLR2/4 and ATP-P_2_X_2_/P_2_Y_7_) on APCs, facilitating antigen uptake and processing. Mature APCs then present tumour antigens to T-cells while concurrently secreting cytokines, such as IL-1β, consequently stimulating T-cell activation and proliferation. Ultimately, cytotoxic T-cells are generated, capable of producing INFγ to eradicate tumour cells. In the meantime, memory T-cells are formed, indicative of the establishment of immunological memory.

Over the last decade, numerous studies have been conducted to discover ICD inducers for therapeutic applications,^38–50^ owing to their ability to directly eradicate cancer cells and concomitantly stimulate adaptive immune responses for tumour eradication. Mechanistically, ICD involves the release of damage-associated molecular patterns (DAMPs) from dying cancer cells.^38,39,50,51^ These DAMPs, including the cell surface translocation of calreticulin (CRT), the extracellular release of high mobility group box 1 (HMGB1), and the extracellular secretion of adenosine triphosphate (ATP), augment the immunogenicity of cancer cells and initiate the cancer-immunity cycle. These processes lead to the recruitment of mature, activated immune cells to the tumour site, ensuring effective antigen capture and presentation (Fig. 1).^38,41,49,50,52^

This review focuses on the unique characteristics of metal complexes and provides an in-depth examination of current metal-based ICD inducers, with a particular emphasis on their molecular targets, mode of mechanism, and roles in enhancing antitumour immune responses. Additionally, we outline the benchmark methods and models employed to validate ICD both *in vitro* and *in vivo*. Unresolved yet significant issues and challenges encountered in the development of metal-based ICD inducers are also discussed. Finally, we conclude this review by highlighting the potential applications of ICD inducers for cancer immunotherapy that could pave the way for future explorations.

## 2. ICD mechanism

ICD is a unique event where cancer cells undergo programmed death while becoming immunogenic and activating adaptive immune response.^26,49^ Consequently, dying cancer cells treated with ICD inducers can be used as a vaccine to prevent tumour proliferation and activate a cancer-specific immune response.^53^ Various cell stressors, including certain traditional chemotherapeutic agents,^27,32,34,54,55^ infective pathogens,^56–59^ and some physical therapeutic modalities, such as photodynamic therapy,^60,61^ extracorporeal photochemotherapy,^62^ electrochemotherapy,^63^ photothermal therapy,^64,65^ radiotherapy,^35,66–68^ high hydrostatic pressure,^69^ and many more,^70–74^ can provoke ICD.

ICD inducers are a type of chemotherapeutic agent that can cause cancer cells to undergo ICD and can be largely divided into two main types: Type I and Type II ICD inducers.^51,73^ The classification of ICD inducers mainly depends on whether they act directly on the endoplasmic reticulum (ER). Type I ICD inducers primarily act on intracellular components other than ER and generate ER stress as secondary or collateral effects. In contrast, Type II ICD inducers target the ER directly, which results in ER stress, thereby initiating ICD.

The ER stress in question typically arises from perturbation in proteostasis and is characterized by an accumulation of misfolded proteins within the ER, which can occur under the influence of ICD inducers.^75–77^ Beyond a tolerable ER stress threshold, an unfolded protein response (UPR) is provoked to restore protein folding capacity. UPR is mediated by three ER stress sensors: protein kinase R-like ER kinase (PERK), inositol-requiring enzyme 1 alpha (IRE1α), and activating transcription factor 6 (ATF6).^78,79^ The PERK pathway, in particular, is crucial for the initiation of ICD.^80,81^ Multiple studies have underscored the importance of ER stress and UPR for ICD induction.^75,77,80^ For example, ER stress was shown to restore the immunogenicity of cisplatin (CDDP)-induced cancer cell death.^82^ Most ICD inducers trigger ER stress for the initiation of the ICD process, with schweinfurthin alkaloids being the notable exception as they can induce ICD without eliciting ER stress.^83^ Moreover, Type II ICD inducers are typically considered more effective than their Type I counterparts, and the ER-targeting strategy has been shown to be an effective approach to reinforce ICD effects.^65,84–86^

In the process of ICD, DAMPs may be surface exposed, released or secreted.^38,50,51^ Most DAMPs are immunologically silent until they are released into the extracellular environment and serve as either adjuvant or danger signals to the immune system. These emitted DAMPs can be recognized by pattern-recognition receptors (PRRs) on immune cells, such as toll-like and nucleotide oligomerization domain (NOD)-like receptors.^87^ The interactions between DAMPs and PRRs facilitate the uptake, processing and presenting of cancer antigens by antigen-presenting cells (APCs), leading to the activation of APCs and T-cells. These activated T-cells then infiltrate into the tumour sites and eradicate the cancer cells (Fig. 1).

### 2.1 Hallmarks of ICD

The induction of ICD is characterized by the emission of DAMPs, namely the translocation of CRT to the outer cell membrane and extracellular secretion of HMGB1 and ATP.^26,88–90^ The concurrent manifestation of these events serves as an indicator of ICD induction *in vitro*. Beyond these classical ICD hallmarks, ICD is also associated with other biological activities, such as cell surface exposure to heat-shock proteins (HSP70 and HSP90)^36,91,92^ and enhanced expression of Type I interferons (IFNs)^93^ and interleukin-1 (IL-1) family cytokines,^94^ which are observed in certain instances.

#### 2.1.1 Calreticulin protein (CRT)

Typically, CRT is a highly conserved soluble protein localized within the ER lumen where it plays a crucial role in maintaining Ca^2+^ homeostasis and acts as a chaperone protein.^95,96^ In the event of ICD, CRT is translocated to the surface of the cell membrane.^31,80,81,97–99^ This relocation process correlates with the phosphorylation of eukaryotic translation initiation factor 2α (eIF2α), an ER stress biomarker, by different kinases (*i.e.* PERK, protein kinase R-PKR and general control nonderepressible 2-GCN2).^80,81,98,100^ Therefore, phosphorylated eIF2α and ER stress are frequently examined in existing studies and considered important ICD signatures.^101–103^ However, it is also noteworthy that eIF2α phosphorylation may not always be necessary for the externalization of CRT.^83,98^ Surface-exposed CRT (ecto-CRT) serves as an “eat me” signal to APCs by interacting with its transmembrane receptor CD91, which is also referred to as low-density lipoprotein receptor-related protein 1 (LRP1).^104^ This interaction stimulates the efficient engulfment of dying cancer cells by phagocytes. Inhibiting ecto-CRT exposure by either knocking down CRT or disrupting its trafficking to the cell surface has been shown to deprive the immunogenicity of dying tumour cells treated with anthracycline.^99^ Conversely, the introduction of exogeneous CRT restores the immunogenicity of non-immunogenic dying cancer cells. Furthermore, growing evidence suggests a link between ecto-CRT and the activation of a robust antitumour immune response.^95,105–107^ Collectively, these findings underscore the importance of ecto-CRT as a pivotal ICD biomarker and its indispensable role in conferring immunogenicity during ICD.

#### 2.1.2 Adenosine triphosphate (ATP)

Other than as an essential intracellular energy supplier for various cellular processes, ATP can be secreted by dying cancer cells into the extracellular environment functioning as signalling molecules.^108–111^ Their release mechanisms in ICD depend on the nature of induction (*e.g.* physical or chemical stress) and treatment duration, involving either pannexin 1 (PANX1)-associated lysosomal exocytosis in an autophagy-dependent manner or passive release at the late stage of cell death.^109,112–116^ Some studies have demonstrated that autophagy plays a significant role in ATP secretion and amplifying ICD effects.^112–114^ Suppressing autophagy by knocking down autophagy-related genes (*e.g.* Atg 5 and Atg 7) reduced the level of secreted ATP upon treatment with MTX and OXP.^112,113^ However, autophagy activation amplifies ICD and improves the chemotherapeutic outcomes of OXP.^117^

Extracellular ATP acts as a “find me” signal for myeloid cells by binding to purinergic receptor P_2_Y_2_ and ionotropic receptor P_2_X_7_, thereby recruiting them to the site of dying tumour cells.^109,111,118^ This promotes their local differentiation and the subsequent effective uptake of tumour antigens *in situ*.^108,110,119^ ATP binding to P_2_X_7_ leads to an efflux of K^+^ and Ca^2+^, which then activate the nucleotide-binding domain, leucine-rich – containing family, and pyrin domain – containing-3 (NLRP3) inflammasomes. This activation drives the secretion of IL-1β, which is essential for the stimulation of IFN-γ producing CD8^+^ T-cells and the tumour-specific adaptive immune system.^118,120,121^ ATP is critical for exerting an effective ICD effect and subsequent immune response activation because depleting extracellular ATP by overexpressing ATPase on the cell surface abolished the immunogenicity of dying tumour cells.^122^ Despite the indispensability of extracellular ATP in ensuring robust anticancer immune response, it is important to note that ATP secretion alone is insufficient for inducing effective ICD, as it may also be present during non-immunogenic cell death.^123,124^

#### 2.1.3 High mobility group box 1 (HMGB1)

HMGB1 is a ubiquitous nonhistone chromatin-binding protein that stabilizes DNA and modulates DNA replication, repair and transcription.^125,126^ However, HMGB1 undergoes changes in subcellular localization and redox state during various cellular stresses, assuming diverse functions as an alarmin protein or a proinflammatory cytokine.^127–135^ For instance, oxidative stress prompts the relocation of HMGB1 to the cytosol, where it facilitates autophagy.^134^ Additionally, HMGB1 is secreted into the extracellular environment in response to different stimuli, where it acts as an immunoadjuvant.^126,127,129,131,132^

In the context of ICD, HMGB1 is passively released at a late stage by dying tumour cells as a “danger” signal.^51,90^ Extracellular HMGB1 can interact with several receptors, including toll-like receptor 2 or 4 (TLR2/4) on DCs, and the receptor for advanced glycation end products (RAGE) to mediate inflammatory responses.^136–138^ The binding between HMGB1 and TLR4 is particularly important for the effective processing and presentation of tumour antigens by DCs, which is essential for the cross-priming of tumour-specific T-cells.^136,137^ The significance of HMGB1-TLR4 interaction has been presented in many studies, showing that the immune responses are compromised through HMGB1 deletion or blockade in chemotherapy-treated dying tumour cells with TLR4 gene disruption.^136,137^ However, it is important to note that the presence of increased extracellular HMGB1 level alone is not indicative of ICD initiation, as it may also be observed when the plasma membrane loses its integrity owing to cell damage.^139^

### 2.2 Identification and validation of ICD inducers

#### 2.2.1 Detection of ICD biomarkers

**Fig. 2:**
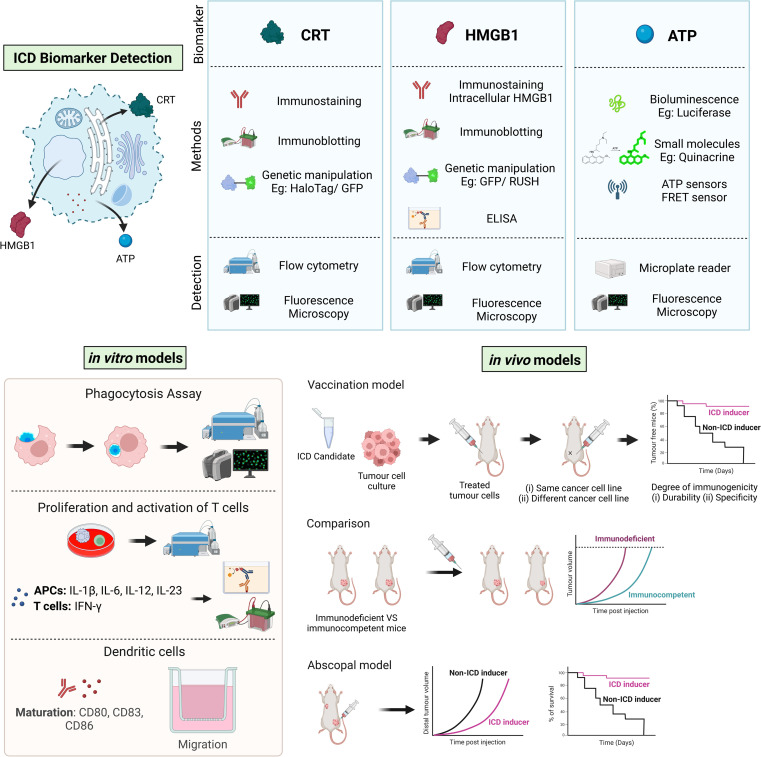
Current screening procedures to identify ICD inducers. Potential ICD candidates are usually screened by determining ICD characteristic biomarkers i*n vitro* using the depicted methods and techniques, followed by *in vitro* immune response activation and, ultimately, *in vivo* vaccination studies.

*In vitro* induction of ICD can be validated through the detection of three primary biomarkers (Fig. 2): the presence of ecto-CRT, the extracellular release of HMGB1, and the extracellular secretion of ATP *via* various assays.^26,88,89^ These assays can be broadly divided into two categories: an indirect approach, which assesses the remaining intracellular levels of these biomarkers, and a direct approach, which quantifies biomarker secretion levels through the direct measurement of secreted components in the extracellular space. For example, the level of ecto-CRT and intracellular HMGB1 can be determined using the immunostaining method with flow cytometry and fluorescence microscopy.^140–142^ However, this method necessitates the use of specific fluorescent antibodies, which can introduce a background signal owing to non-specific binding. Similarly, immunoblotting is also widely used in the determination of ecto-CRT and intracellular HMGB1 levels.^31,36,143^ The levels of intracellular HMGB1 then provide an indirect measurement of its corresponding extracellular levels owing to an assumed reverse relationship between the two. Moreover, extracellular ATP secretion can be indirectly determined using ATP-sensitive fluorophore, quinacrine, which enables the quantification of intracellular ATP levels in the residual ATP pool only if the ICD candidates do not target energy metabolism.^144–146^

In contrast to measuring the residual pool of intracellular HMGB1 indirectly, extracellular HMGB1 can be directly quantified *via* enzyme-linked immunosorbent assay (ELISA).^32,69,147^ Likewise, extracellular ATP can be directly quantified by bioluminescent ATP detection assay, in which ATP is consumed by luciferase enzyme to catalyse the light-emitting oxidation of luciferin.^145,148^ However, this direct measuring method can be confounded by expression of ATP degrading enzymes, such as CD39, in some cell lines.^122^

Despite the reliability of these assays, they are generally time-consuming and tedious. Therefore, to accelerate the discovery of new ICD inducers, several platforms for screening ICD biomarkers have been established.^34,82,141,142,149–151^ Particularly, researchers have engineered human osteosarcoma U2OS cells with diverse visualizable or detectable indicators, such as CRT-GFP chimera,^34^ CRT-HaloTag fusion protein,^82,152^ HMGB1-GFP chimera,^34,142^ HMGB1-SBP-GFP (SBP, streptavidin-binding peptide),^141^ and implemented ATP-specific fluorescence resonance energy transfer (FRET)-based reporters,^34,145,151^ for high throughput screening (879 candidates included in the NCI Mechanistic Diversity Set) at different concentrations.^149^

#### 2.2.2 *In vitro* models

Although the detection of DAMPs is a standard approach for investigating ICD inducers, it alone is insufficient to demonstrate an effective ICD-primed immune response owing to the intricacy of intracellular pathways that affect the immunogenicity of tumours. To examine whether ICD candidates can elicit immune responses *in vitro*, ICD-succumbing cancer cells or their culture supernatants can be exposed to immune cells, predominantly APCs and T-lymphocytes (Fig. 2).^32,69,153^ This is followed by a series of functional assays to evaluate (1) the phagocytic capacity of phagocytes to engulf damaged cancer cells and their debris; (2) the maturation, migration, and ability of APCs to stimulate cross-presentation of cancer antigens to T-cells; and (3) the proliferation and activation of T-cells.

The engulfment of dying cancer cells and their corpses can be assessed by phagocytosis assay.^54,154–158^ In this assay, mononuclear phagocytes (*e.g.* macrophages, monocytes) and cancer cells are labelled separately using non-toxic fluorescent dyes or expression of different reporter fluorescent proteins, and co-cultured after the cancer cells are treated with ICD candidates. Phagocytes with engulfed treated tumour cells exhibit dual fluorescence signals, which can be subsequently quantified by flow cytometry or fluorescence microscopy. The percentage of phagocytes with dual fluorescence emissions represents the degree of phagocyte activation. Subsequently, the co-culture experiment could be repeated in the presence of a CRT-specific antibody or CRT-binding peptide to block the interaction of the phagocytes with the treated tumour cells.^99^ A statistically significant reduction in phagocyte activation would implicate CRT in the phagocytic response, as expected in ICD induction, and rule out other non-specific causes. Markers of DC maturation, such as CD80, CD83, and CD86, can be detected using immunostaining techniques.^32,69,159^ The migratory ability of these cells is often assessed using a *trans*-well migration assay.^160^

The proliferation and activation of T-cells can be assessed by isolating T-cells that have been co-incubated with cancer cells and subsequently analyzed *via* flow cytometry.^26,161^ Meanwhile, the profiling of cytokines in the supernatant, such as IL-1β, IL-6, IL-12, and IL-23 produced by APCs, or IFN-γ by T-cells, can be conducted post-coculture to appraise the activation status of the immune cells.^32,69^ Cytokine levels are then measured using specific ELISA kits or flow cytometry. These comprehensive *in vitro* approaches allow for a detailed understanding of the immune response elicited by potential ICD inducers.

#### 2.2.3 *In vivo* models

The ICD induction capability of potential candidates should be functionally evaluated using appropriate murine models. Currently, several *in vivo* models are in use, among which the *in vivo* vaccination model being the gold standard (Fig. 2).^26,53,89,162,163^ This model evaluates the potential of treated tumour cells as a type of cancer vaccine to prevent future tumour development in immunocompetent mice. In a typical procedure, cancer cells succumbing to treatment by an ICD inducer are subcutaneously injected into the flanks of immunocompetent mice. After a period of one to two weeks, the mice are then rechallenged with viable cancer cells into the opposite flank. This is followed by routine monitoring for any signs of tumour formation and growth. The proportion of mice that remains tumour-free and the rate of tumour growth upon cancer cell rechallenge indicate the degree of immunogenicity of cancer cells treated with potential ICD candidates. Ultimately, tumour-free mice could be subjected to a second rechallenge with the same cancer cell line to assess the durability of tumour prevention. To ascertain specificity, a rechallenge with another synergistic cancer cell line may also be conducted.

Another approach that is also being explored as a viable cancer vaccine strategy is to use DCs that have been exposed to ICD-succumbing cancer cells instead.^164–166^ Complementary assessment and comparison of tumour growth and immune response in immunocompetent and immunodeficient mice can be conducted to verify the role of the immune system in tumour prevention. Finally, the abscopal response model is used as an alternate model to validate ICD inducers.^167–170^ For the abscopal response model, two lesions (*i.e.* primary tumour and secondary tumour) are generated at two different sites in mice. The primary tumour is subjected to localized treatment, while the secondary or distant tumour is monitored for any signs of tumour growth and metastasis, which would indicate the induction of ICD.

## 3. Metal complexes as ICD inducers

**Fig. 3:**
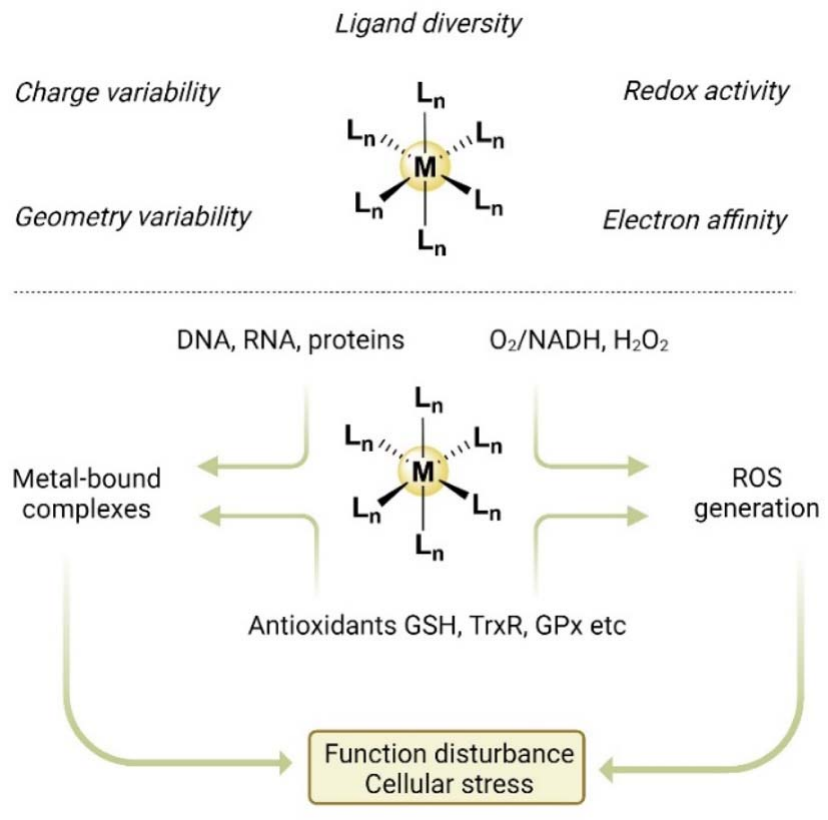
The unique properties of metal complexes and potential modes of action for ICD action.

Inorganic metal complexes exhibit unique characteristics that stem from the varied interactions between metallic and non-metallic elements (Fig. 3).^171–173^ First, metal-containing molecules are usually positively charged, but the overall complexes can be cationic, anionic or neutral depending on their associated ligands and counterions. The overall charge of a metal complex can significantly influence its biological activities and therapeutic outcomes. Second, metal ion centers can chelate with diverse ligands and display distinct coordination geometry. Structural modification can be achieved simply by replacing ligands, giving rise to a wide variety of inorganic complexes with markedly distinct activities. Third, metal ions possess different oxidation states that can readily interconvert through redox processes.^174–176^ Hence, under physiological conditions, metal complexes can disrupt intracellular redox balance through several possible mechanisms: (1) directly initiating ROS generation *via* Fenton reaction (M^*n*+^ + H_2_O_2_ → M^(*n*+*z*)+^ + OH^−^ + ˙OH) as catalysts, such as Fe, Cu, Co, Mn, Ag, and Ru;^177^ (2) producing H_2_O_2_ by catalyzing hydride transfer from NADH to oxygen;^178^ (3) interacting with intracellular antioxidants, such as glutathione (GSH), thioredoxin reductases (TrxR), and glutathione peroxidases (GPx) owing to their nucleophilicity and high electron affinity.^174–176,179–181^ These modes of action confer potent cytotoxicity to metal complexes in combating neoplastic cells.

Ideally, an ICD inducer should activate multiple ICD-associated pathways to ensure robust and effective ICD effects. Metal-based complexes represent a class of compounds that have the potential for this capability owing to their diverse modes of action. These include not only their binding affinity to DNA but also to a multitude of proteins, which can effectively cause cellular stress and physiological disturbance. The capacity of metal-based agents to disturb intracellular redox balance stands out in the search quest for ICD inducers. This is because cellular stress induced by reactive oxygen species (ROS), especially ROS-mediated ER stress, is highly associated with ICD induction.^75–77,82,182^ Furthermore, beyond their direct cytotoxic effects, accumulating evidence supports the importance of certain metal-based agents in promoting ICD-driven antitumour immunity.^183–186^

To date, a wide variety of metal-based ICD inducers have been discovered with different metals, including platinum (Pt), iridium (Ir), gold (Au), ruthenium (Ru), copper (Cu), rhenium (Re), and manganese (Mn). Most of them can be classified as Type II ICD inducers. For ease of reference, we categorize these metal-based ICD inducers based on their metal centers, followed by their coordination chemistry. We further examine their efficacies and activities using reported *in vitro* and *in vivo* results and study their design strategies. We discuss their molecular targets and mechanisms of action, where applicable, and consider their potential application for cancer therapy.

### 3.1 Pt-based ICD inducers

#### 3.1.1 OXP and its Pt(ii) derivatives

**Fig. 4:**
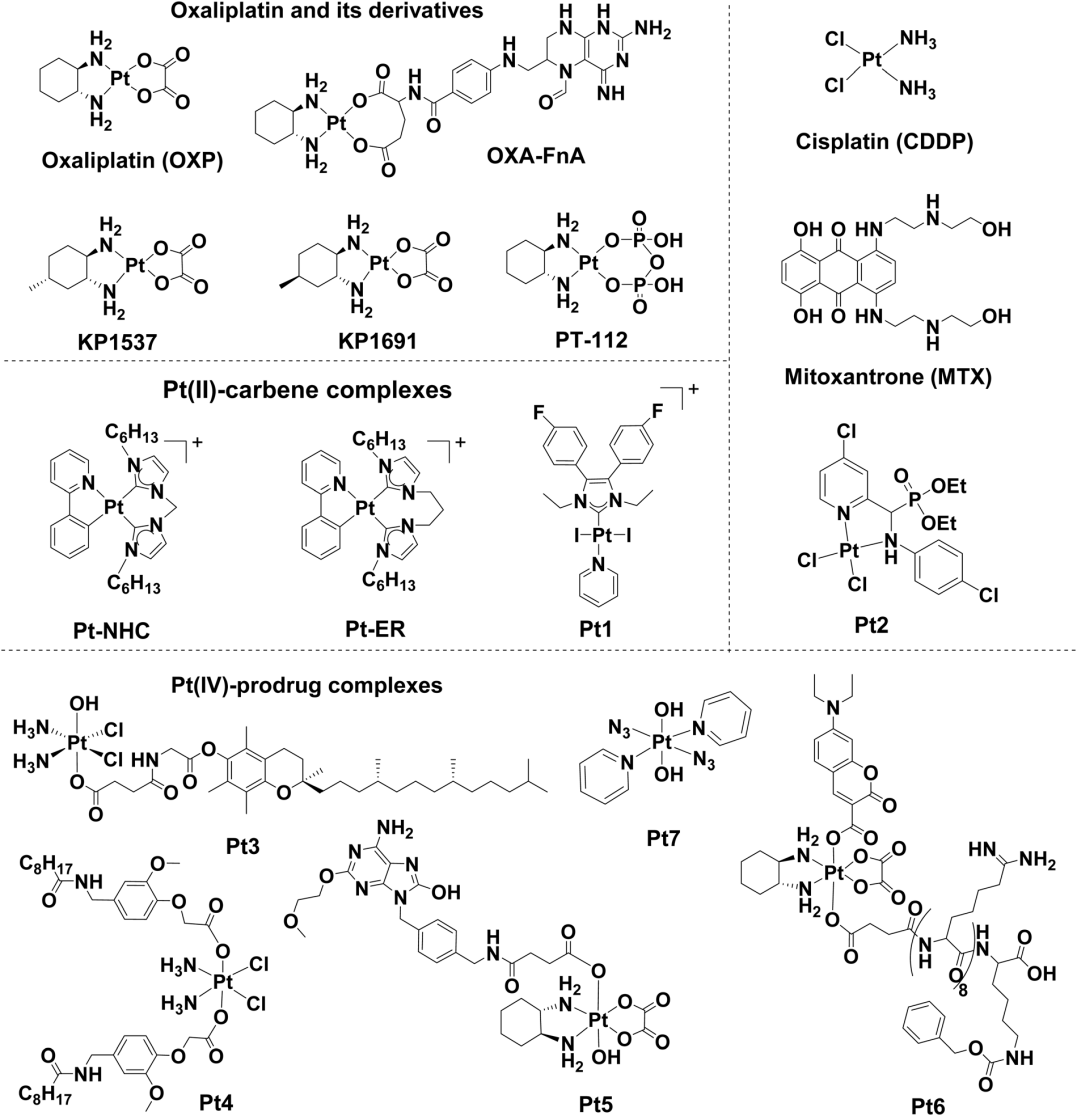
Molecular structures of reported Pt-based ICD inducers. Counter anions are omitted for clarity.

In 2010, OXP (Fig. 4) was reported to induce ICD in colon cancer cells by causing pre-apoptotic CRT exposure and HMGB1 release, thereby stimulating the antitumour immune response in immunocompetent mice implanted with CT26 tumours.^33^ In contrast, CDDP was able to efficiently induce HMGB1 release but it failed to trigger CRT exposure and subsequent anticancer immune response. This finding established a connection between the therapeutic efficacy of OXP in colorectal cancer and ICD for the first time. Although OXP can bind DNA as the primary target, in keeping with CDDP, OXP might interfere with other biological processes that cause cellular stress, leading to ICD induction. OXP is thus widely regarded as a Type I ICD inducer based on its role in causing ribosomal biogenesis stress.^187^ The ability of OXP to induce ICD was further described in various cancer models, such as laryngeal cancer,^188^ lung carcinoma^189^ and hepatocellular carcinoma.^190^ Currently, there are several ongoing studies to investigate OXP in clinical settings. A Phase II trial (NCT00126256) first found that, compared with 5-fluorouracil (5-FU) alone, 5-FU in combination with OXP improved progression-free survival and overall survival. Additional cases of OXP-induced ICD for cancer treatment are presented in Table 1.

**Table 1:** List of ongoing trials for OXP and PT-112 for cancer immunotherapy

Trial title	Treatment	Indication	Phase	Status	Reference
**OXP-based clinical trials**					
Study of S 95005 in combination with oxaliplatin in metastatic colorectal cancer	OXP + trifluridine + bevacizumab + nivolumab	Metastatic colorectal cancer	I	Completed	NCT02848443
Dendritic cell vaccine and chemotherapy for patients with pancreatic cancer (PancVax)	OXP + FA + irinotecan + 5-FU + PTX + gemcitabine + a DC-based vaccine	Pancreatic cancer	I	Terminated	NCT02548169
Nivolumab (anti-PD1 antibody) and ipilimumab (anti-CTLA4 antibody) in combination with immunogenic chemotherapy for patients with advanced non-small cell lung cancer	OXP + nivolumab + ipilimumab	Advanced NSCLC	II	Active	NCT04043195
Chemotherapy and immunotherapy as treatment for MSS metastatic colorectal cancer with high immune infiltrate (POCHI)	OXP + capecitabine + bevacizumab + pembrolizumab	Metastatic colorectal cancer	II	Recruiting	NCT04262687
METIMMOX: colorectal cancer metastasis – shaping anti-tumour immunity by oxaliplatin (METIMMOX)	OXP + 5-FU + leucovorin + nivolumab	Metastatic colorectal cancer	II	Active	NCT03388190
Safety and efficacy of pembrolizumab (MK-3475) in combination with TS-1 + CDDP or TS-1 + oxaliplatin as first line chemotherapy in gastric cancer (MK-3475-659/KEYNOTE-659)	OXP + pembrolizumab or CDDP + TS-1	Gastric cancer	II	Completed	NCT03382600
Rectal artery infusion chemotherapy of oxaliplatin plus capecitabine combined with anti-PD1 antibody after induction chemotherapy for microsatellite stable locally advanced rectal cancer: a prospective single-arm phase II study	OXP + rectectomy + capecitabine + anti-PD-1 monoclonal antibody	Advanced rectal cancer	II	Recruiting	NCT05307198
Neoadjuvant arterial embolization chemotherapy combined PD-1 inhibitor for locally advanced rectal cancer (NECI)	OXP + capecitabine + tislelizumab	Rectal cancer	II	Recruiting	NCT05420584
METIMMOX-2: metastatic pMMR/MSS colorectal cancer – shaping anti-tumour immunity by oxaliplatin (METIMMOX-2)	OXP + nivolumab	Metastatic colorectal cancer	II	Recruiting	NCT05504252
Trial comparing two strategies of chemotherapy for metastatic colorectal cancer	OXP + 5-FU + irinotecan + leucovorin	Colorectal cancer	III	Completed	NCT00126256

**PT-112-based clinical trials**					
A phase 1/2a dose-finding study of PT-112 in patients with relapsed or refractory multiple myeloma	PT-112	Multiple myeloma	I	Completed	NCT03288480
PT-112 in subjects with thymoma and thymic carcinoma	PT-112	Thymoma and thymic carcinoma	II	Recruiting	NCT05104736
A study evaluating the safety, pharmacokinetics, and clinical effects of intravenously administered	PT-112	Thymoma and thymic carcinoma; metastatic castrate-resistant prostate cancer	II	Active	NCT02266745
PT-112 injection in subjects with advanced solid tumours and subsequent dose expansion cohorts					
An open-label phase I/II clinical trial of PT-112 injection for advanced solid tumours and advanced hepatocellular carcinoma	PT-112	Hepatocellular carcinoma (HCC)	II	Unknown	NCT03439761
An open-label phase I/II clinical study of PT-112 in combination with docetaxel in subjects with advanced solid tumour in a phase I dose escalation study and in subjects with non-small cell lung cancer (NSCLC) in a phase II dose confirmation study	PT-112 + docetaxel	Advanced solid tumours and NSCLC	II	Unknown	NCT02884479
PT-112 (phosplatin's platinum) combined with gemcitabine injection for advanced solid tumours	PT-112 + gemcitabine	Biliary tract cancer	II	Unknown	NCT05357196
A dose escalation and confirmation study of PT-112 in advanced solid tumours in combination with avelumab (PAVE-1)	PT-112 + anti-PD-L1 antibody (avelumab)	NSCLC	II	Completed	NCT03409458

The discovery of OXP as a potent ICD inducer fuels further research into optimizing OXP-based cancer chemo-immunotherapy with enhanced antitumour efficacy, developing novel Pt-based ICD inducers and understanding the underlying associated molecular mechanisms. For example, several OXP-based macromolecules combined with immune checkpoint inhibitors (ICIs) or other immuno-modulatory agents have been constructed with the potential to reverse immunosuppressive TME, minimizing undesirable adverse effects and boosting immunotherapeutic efficacy.^191–199^ Compared to OXP alone, these nanostructures generally exhibited higher cytotoxicity, lower side effects and superior antitumour immunity. One such example is the nano-FOLFOX delivery system that released [Pt(DACH)(H_2_O)_2_]^2+^, the active form of OXP, and folinic acid (FnA) to form OXP-FnA adducts that could inflict ICD and exhibit anticancer activity.^200^

Given the role of the 1*R*,2*R*-diaminocyclohexane (DACH) ligand in the activity of OXP, a few studies have explored the relationship between ligand structures and ICD-inducing activity. A study in 2012 suggested that adding methyl groups to DACH ligands (KP1537 and KP1691) could influence its side effects and ICD-inducing capacity.^201^ Interestingly, in contrast to OXP, which elicited ICD-driven antitumour immune response only in immunocompetent but not immunocompromised mice, KP1537 and KP1691 exhibited anticancer activity in both types of mice. Another study investigated the abilities of distinct diaminocycloalkanes chelating OXP analogues to induce ICD-associated DAMPs emission.^202^ Efforts to achieve the optimal structural modifications of the DACH ligand motif to yield new OXP analogs may be valuable for the development of new ICD inducers.

PT-112 is the subject of several ongoing clinical investigations in patients with solid tumours and hematologic malignancies, either as monotherapy (NCT02266745) or in combination therapy with PD-L1 inhibitor avelumab (NCT03409458). PT-112 was developed as a novel ICD inducer by replacing the oxalate ligand in OXP with a pyrophosphate group.^203–205^ Although PT-112 shares structural similarity with OXP as it retained the Pt-(DACH) pharmacophore (Fig. 4), the pyrophosphate moiety not only enhanced its pharmacokinetic and pharmacodynamic properties but also reduced its toxicity. PT-112 exerts its cytotoxicity on cancer cells through different mechanisms of action from traditional DNA-damaging Pt agents. In a murine breast carcinoma TSA cell model, PT-112 was compared with a well-established organic small molecule ICD inducer MTX and showed superior ability to stimulate immunostimulatory DAMP-accompanied ICD induction and the establishment of long-term immunologic anticancer memory *in vivo*.^203^ Impressively, in a vaccination model, PT-112-treated breast carcinoma TSA cells conferred 100% immunological protection against the subsequent injection of living TSA cells. Anti-tumour protection exhibited good durability when the mice were rechallenged after 60 days. PT-112 combined with ICBs achieved tumour elimination, enhanced cytotoxic T lymphocytes (CTLs) infiltration and reduced immunosuppressive CD25^+^FOXP3^+^ regulatory T-cells (Tregs) and tumour-associated macrophages (TAMs) in the TME. These results demonstrated that PT-112 exhibited remarkable therapeutic efficacy in eradicating tumour and establishing long-term antitumour immunity. Several Phase I/II trials for PT-112 as a monotherapy (NCT05104736, NCT02266745, NCT03288480, and NCT03439761) and in combination with the PD-L1 inhibitor avelumab (NCT03409458) in treating immunologically “cold” advanced metastatic castration-resistant prostate cancer, or with other chemotherapeutic agents, including docetaxel (NCT02884479) and gemcitabine (NCT05357196), have yielded promising results. Ongoing trials investigating PT-112-induced ICD for cancer treatment are shown in Table 1.

#### 3.1.2 CDDP in combination treatment

Distinct from other chemotherapeutic agents, such as doxorubicin, cyclo-phosphamide, bortezomib, and paclitaxel, CDDP alone cannot induce ICD and subsequent protective antitumour immune response.^33^ This has been attributed to a failure in triggering ER stress-associated phosphorylation of eIF2α when CDDP was treated alone. Nevertheless, when CDDP was used in conjunction with ER stress inducers, such as thapsigargin or tunicamycin, CRT cell surface exposure and the immunogenicity of treated cancer cells can be reestablished.^82^ In addition to ER stress inducers, Type I IFN was proven to be effective in restoring the phosphorylation of eIF2α and CRT surface exposure in a sequential interferon β (IFN-β) and CDDP treatment^93,206^ although whether this combination therapy can also enhance the antitumour immune response *in vivo* remains unclear. The effectiveness of IFN-β-CDDP sequential treatment was likely to be related to the release of chemokine (C–X–C motif) ligand 10 (CXCL10) *via* IFN-β-triggered autocrine and paracrine circuitries.^93^CDDP alone cannot stimulate Type I IFN release.

#### 3.1.3 Pt(ii)-carbene complexes

In 2015, the first systematic study was initiated by Ang group on the ICD-inducing ability of some chemotherapeutically active Pt agents, such as CDDP, OXP, carboplatin, picoplatin, satraplatin, phenantriplatin and newly discovered preclinical Pt agents.^207^ An ER-targeting cyclometalated complex Pt-NHC with a unique scaffold was found to be an effective Type II ICD inducer characterized by ER stress induction, classical ICD hallmarks emission and CRT-dependent phagocytosis. Pt-NHC was first reported by the Che group as an ER-specific dye and was previously shown to accumulate in the ER, causing ER stress and apoptosis.^208^ Unlike other Pt agents, it preferentially binds to proteins rather than DNA. Following this discovery of its ICD induction ability, an extensive structure–activity relationship study analysis was conducted, leading to the discovery of Pt-ER with improved properties.^209^ Similar to Pt-NHC, Pt-ER was also a Type II ICD inducer that triggered ICD *via* ROS-driven ER stress. However, Pt-ER exhibited more pronounced ICD-associated DAMPs emission and phagocytosis compared to Pt-NHC. In addition, the effectiveness of ICD-inducing Pt-NHC in improving chemoimmunotherapy was further corroborated by other studies. For example, Pt-NHC-containing nanoparticles displayed a superior ability to eradicate triple-negative breast cancer tumour and enhance overall survival in mice.^210^ When combined with Interleukin-2, Pt-NHC-based nanogel reprogramed the immunosuppressive TME in “immunologically cold” tumours, *i.e.* markedly reduced TAMs and the infiltration of Tregs, including pancreatic ductal adenocarcinoma (PDAC) and hepatocellular carcinoma (HCC).^211^

In addition to the cyclometallated Pt scaffold examples, other Pt(ii)-carbene complexes have been explored for their capacities for evoking ICD in HCC. Liu group designed and evaluated a series of Pt(ii)-carbene complexes derived from 4,5-diarylimidazole. One of 19 compounds, Pt1, triggered DAMPs emission and anti-HCC immune response.^212^ In a follow-up study, replacing iodide ligands with other halogen atoms did not affect the ICD-inducing capacity of the 4,5-diarylimidazole-based Pt(ii)-NHC scaffold, indicating the importance of carbene ligands.^213^ Notably, the reported Pt-carbene complexes were Type II ICD inducers and triggered ROS-associated ER stress-driven ICD, suggesting the high relevance of carbene ligands in the development of novel Pt-based Type II ICD inducers.

#### 3.1.4 Pt(ii) compounds with other ligands

A aminophosphonate-chelating Pt(ii) complex Pt2 was identified as a *bona fide* Type II ICD inducer associated with oxidative ER stress out of 11 purposefully designed analogs with different substituents (Cl, H, and OMe) on the aminophosphonate-pyridine ligand.^214^Pt2, bearing two Cl substituents, showed the highest cytotoxicity and elicited DMAP signals *in vitro*, as well as induced anti-tumour immune response *in vivo*. A supramolecular construct, self-assembled by Pt(ii) metallacycle and an aza-dipyrromethene boron difluoride (aza-BODIPY) ligand to yield a triangular hexanuclear Pt(ii) complex, was identified as a potent ICD inducer targeting lysosome.^215^ Upon near-infrared (NIR) light excitation, the Pt(ii) metallacycle-based supramolecule triggers significant ROS production in deep-seated tumours, as well as ICD-based antitumour immune response in vaccinated mice.

#### 3.1.5 Pt(iv) prodrug complexes

Although Pt(ii) anticancer agents have been highly successful in the clinical treatment of various solid tumours, the development of Pt(v) prodrugs is emerging as a strategy to alleviate their significant drawbacks, including severe undesirable side effects and the emergence of drug resistance. Additionally, extra axial ligands available offered a way to alter the chemical and biological properties of Pt(iv) prodrugs.^216,217^ One example is the enhancement of the immunomodulatory properties of CDDP through the design of CDDP-based Pt(iv) prodrugs with bioactive ligands that could facilitate ICD induction because CDDP alone cannot induce ICD. For instance, a tocopherol-conjugated Pt(iv) complex, Pt3, delivered intratumourally through hyaluronan (HA)–tocopherol nanocarriers, stimulated CRT translocation to the cell surface,^152^ as observed in AT84 cells overexpressing a mouse CRT-HaloTag-KDEL fusion protein. Wang *et al.* purposefully constructed a CDDP-based Pt(iv) complex Pt4 by installing an ICD-inducing molecule, capsaicin, as axial ligands *via* carboxylic functionalities.^218^ Compared to capsaicin, Pt4 strengthens ICD effects and promotes phagocytosis by THP-1-derived macrophages and secretion of IFN-γ and TNF-α from human peripheral blood mononuclear cells (hPBMCs).

As previously discussed, toll-like receptors (TLRs) are essential pattern recognition receptors on DCs and macrophages for recognizing ICD-associated DAMPs and initiating an immune response.^136–138^ Given the important role of TLRs, Wang group fabricated OXP-based Pt(iv) prodrug Pt5 conjugated with a TLR7 agonist.^219^ As expected, in addition to instigating CRT translocation and ATP secretion, Pt5 promotes DC activation *in vitro* characterized by enhanced secretion of proinflammatory cytokines IFN-γ, TNF-α, IL-6, and IL-12 and clearly increased percentages of intratumourally infiltrated CD8^+^ T-cells *in vivo* compared to OXP or the TLR7 agonist itself.

In contrast to activation by reductants in the aforementioned studies, a photoactivatable Pt(iv) prodrug complex Pt6 bearing coumarin axial ligands induced ICD upon photoirradiation.^220^Pt6 exhibited superior phototoxicity towards multiple cell lines, including two CDDP-resistant ones. Upon photoactivation of Pt6, distinct ICD biomarkers were observed in the treated A549cisR cells. In contrast, no detectable ICD effects were observed upon Pt6 treatment in the dark. Pt6-treated A549cisR cells largely promoted T-cell proliferation in mixed leukocyte reactions. Another photoactivatable Pt(iv)-azido prodrug Pt7 was capable of inducing ROS and reactive nitrogen species (RNS) production and, simultaneously, releasing cytotoxic Pt(ii) species.^221^ Under blue light irradiation, Pt7 induces autophagic cell death accompanying 3 characteristic ICD signatures and promotes phagocytosis of Pt-treated CT26 carcinoma cells by J774.A1 macrophages as well.

### 3.2 Ir-based ICD inducers

#### 3.2.1 Ir(iii)-polypyridyl complexes

**Fig. 5:**
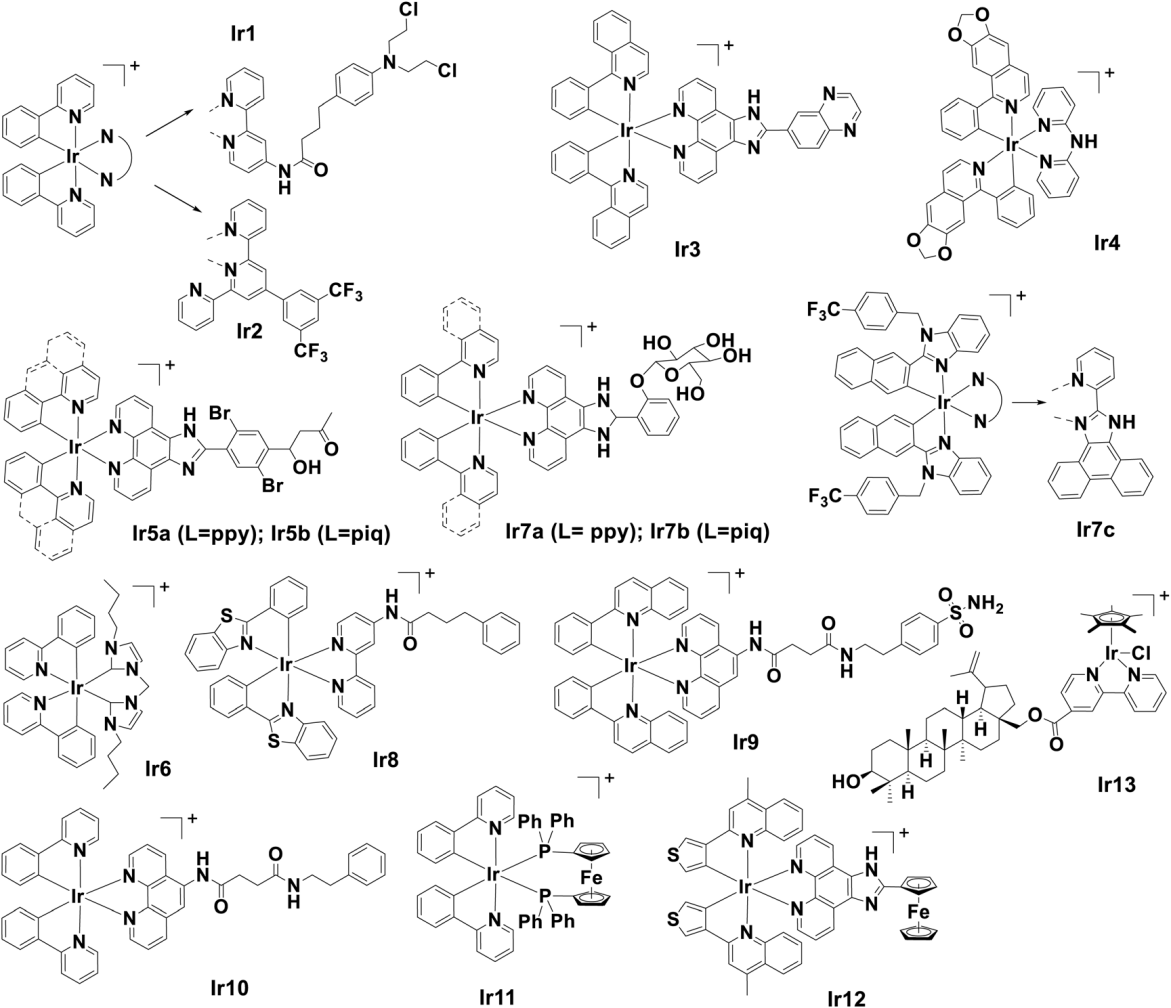
Molecular structures of reported Ir-based ICD inducers. Counter anions are omitted for clarity.

Ir complexes bearing various modifiable ligands have been exploited as therapeutic agents in cancer diagnosis and treatment. Ligands of Ir complexes influence their subcellular localization, activity, and mechanism of action accompanied by different cell death modes. Recent studies have highlighted the potential of multiple Ir(iii)-polypyridyl complexes for apoptotic, paraptotic or ferroptotic ICD. Chao *et al.* reported an ER-targeting Ir(iii) complex, Ir1 (Fig. 5), that induced ICD *via* apoptosis in non-small-cell lung cancer (NSCLC).^222^Ir1 triggered ER stress, which led to the release of Ca^2+^, mitochondrial dysfunction, and ROS overproduction, culminating in apoptosis *via* a caspase-dependent pathway. This was evidenced by increased caspase 3/7 activation. In particular, the vaccination assay conducted *in vivo* demonstrated that tumour volume was 4.59-fold smaller than the control and that the ratio of immunostimulant cytotoxic T-cells (CD8^+^) against immunosuppressive Foxp3^+^ T-cells was 4.9-fold higher. Another ppy-based Ir(iii) complex, Ir2, with fluorinated tridentate derivative was also reported to accumulate in the ER and induce ROS-driven ERS-based ICD *via* apoptosis.^223^ Notably, evidence suggested that Ir2 can enhance the anti-tumour immunity of PD-1 inhibitors in a poorly immunogenic B16-F10 melanoma model *in vivo*. Although Ir2 or PD-1 treatment alone can reduce tumour growth, the combination of the Ir2 and PD-1 groups displayed the most pronounced tumour suppression during the 12-day study duration. An increase in the ratio of CD8^+^ T-cells and Foxp3^+^ T-cells in tumour tissue further confirms the remodeling of the TME. Cyclometalated Ir(iii) complex Ir3 undergoes ER stress and paraptosis to induce DAMPs in HepG2 cells.^224^ Interestingly, no ROS generation was required for ICD induction of such cell death. Flow cytometry using the fluorescent probe 2′,7′-dichlorodihydrofluorescein diacetate showed that the intracellular ROS levels decreased to a level lower than that of the control group when the concentration of Ir3 increased.

Recently, Liang *et al.* reported cyclometalated Ir(iii) complex Ir4 based on isoquinoline alkaloid-induced autophagy-dependent ferroptosis and ICD response in triple-negative breast cancer (TNBC) cells, thereby triggering the emission of DAMPs.^225^Ir4 exerted its cytotoxic effect *via* the generation of ROS that induced ferroptosis and downregulation of indoleamine 2,3-dioxygenase (IDO), an immunosuppressive enzyme. It also activated CD8^+^ T-cells and reduced regulatory T-cells (Tregs). Ir4 showed superior efficacy compared to traditional chemotherapy agents, such as OXP. Similar to Ir2, the combination of Ir4 with anti-PD1 therapy significantly improved tumour inhibition.

A recent study showcased two Ir(iii) complexes, Ir5a and Ir5b, as effective inducers when they were delivered to the ER using a liposome-based encapsulation strategy.^226^ Liposomal encapsulation greatly enhanced the cellular uptake of Ir5a and Ir5b, which preferentially accumulated in the ER and triggered oxidative stress and apoptotic ICD. Without facilitated delivery, Ir5a and Ir5b alone exhibited weak cytotoxicity and CRT exposure owing to low cellular uptake efficiencies. Despite the further structural optimization required, this study demonstrated the versatility of carrier-aided strategies to enhance ICD effects.

Another study by Zou group reported an ER stress-inducing cyclometalated Ir(iii)-bis NHC complex (Ir6) that can elicit ICD hallmarks both *in vitro* and *in vivo* using the vaccination model.^227^ The innovative use of a specially designed clickable photoaffinity probe showed that Ir6 could directly bind with and subsequently inhibit BiP, a key regulator of the UPR pathway that functions as a protein chaperone, aiding in protein proper folding and assembly.^228^ This work was significant as it was the first time that the molecular target of an ICD inducer was systematically uncovered using chemical biology approaches.

#### 3.2.2 PDT-based Ir(iii) complexes

The unique photophysical properties of Ir(iii) complexes make them highly suitable as photosensitizers for photodynamic therapy (PDT).^229^ In this approach, Ir(iii)-based PDT agents continuously induced ROS through photoirradiation to exert ER stress, triggering ICD. The photocatalytic performance and therapeutic effects could be fine-tuned by modifying phenylpyridyl ligands. Multiple Ir(iii) complexes, such as Ir(iii) with phenylpyridine backbone (ppy) (Ir7a) and phenylisoquinoline (piq) (Ir7b)^230^ or modified imidazole (Ir7c),^231^ demonstrated high effectiveness as photosensitizers for PDT and induced ICD upon irradiation. In particular, Ir7c selectively targeted cancer stem cells (CSCs).

Ir-pbt-Bpa Ir8 was developed for two-photon excitation photodynamic immunotherapy by replacing the ancillary ligand 2-phenylpyridine with 2-phenylbenzo[*d*]-thiazole.^232^ This modification enhanced two-photon absorption, increased ROS production, and shifted the primary subcellular target from the ER to the mitochondria, leading to cell death in melanoma cells *via* ferroptosis. This stress response was enhanced by Ca^2+^ release from the ER, resulting in significant detection of ICD biomarkers and a significant reduction of both primary and distant melanoma tumours, even though only the primary tumour was directly treated. Histological examinations showed enhanced DC maturation and inhibition of tumour immunosuppression, as indicated by a favorable CD8^+^/Foxp3^+^ ratio. This study highlighted the significance of ligand modification in influencing their subcellular localization and biological activity, and the importance of targeting other organelles such as mitochondria, in addition to ER, for inducing ICD.

The incorporation of the carbonic anhydrase IX (CAIX)-targeting group into phenylpyridine-, difluorophenylpyridine-, and phenylquinoline-based Ir(iii) complexes was investigated as an approach for the treatment of HT29 colon cancer cells *via* PDT.^233^ Upon irradiation with light (*λ*_ex_ at 425 nm), phenylquinoline-based Ir9 induced pyroptosis under hypoxic conditions. Ir9 targeted CAIX, an enzyme highly expressed in hypoxic tumours, leading to its degradation. This, in turn, downregulated the expression of hypoxia-inducible factor 1α (HIF-1α) levels and vascular endothelial growth factor (VEGF) expression, improving the cancer immune microenvironment.

Another Ir(iii) photosensitizer Ir10 with a phenanthroline ligand modified with a hydrophobic long-chain ER targeting *N*-phenethylsuccinamide moiety generated ROS upon irradiation in oral squamous cell carcinoma (OSCC), which elicited ER stress, leading to ICD and an upregulation of PD-L1 expression.^234^ The combination with PD-L1 inhibitor was particularly effective in converting “cold” tumours (with low immune activity) into “hot” tumours (with high immune activity) *in vivo*. The combination significantly upregulated the level of mature DCs (MHC II^+^ and CD80^+^CD86^+^ DCs), T-cell infiltration (CD4^+^ and CD8^+^) and cytokines (TNF-α and IFN-γ) while downregulating immunosuppressive inflammatory cytokine IL-6, signifying transformation into “hot tumour”.

#### 3.2.3 Other Ir(iii) complexes

The incorporation of redox-active functional groups could be a strategy to develop ferroptosis inducers by imparting Ir(iii) complexes with the ability to catalyze a Fenton-like reaction, generating hydroxyl radicals and lipid peroxidation. Mao and Tan group incorporated the ferrocene moiety to generate Ir(iii) complexes containing ferrocene to induce ferroptosis-coupled ICD, subsequently enhancing cancer immunity.^235^ In particular, cyclometalated ppy-based Ir(iii) complex Ir11 containing a ferrocene-modified diphosphine ligand was reported to induce ICD, characterized by DAMPs emission. A significant inhibition rate in primary and distal tumours was observed, with a 2-fold increase in CD8^+^ T-cells in distal tumours. Similarly, the same group reported a Type I Ir(iii) photosensitizer with ferrocene moiety, Ir12, which also induced ferroptosis to initiate ICD.^236^ Upon light activation at 425 nm, Ir12 was able to induce the 3 biomarkers *in vitro* in MDA-MB-231 cell lines and enhance the activation of cytotoxic T-cells and maturation of DC cells in the abscopal response model.

A cyclopentadienyl Ir(iii) complex with natural product betulin, Ir13, activated the ferroptosis cascade through ferritinophagy and iron homeostasis regulation.^237^Ir13 activated PERK/eIF2α pathway and CRT exposure, and HMGB1 and ATP release were detected *in vitro* in A549 cancer cells. RNA sequence analysis indicated that ferroptosis and nuclear factor kappa light chain enhancer of activated B cells (NF-κB) activation further amplified the antitumour effect. The *in vivo* vaccination model showed that Ir13 inhibited tumour growth and upregulated the expression of proinflammatory cytokines and cytotoxic T-cells to stimulate a robust immune response.

### 3.3 Au-based ICD inducers

#### 3.3.1 Au(i)-phosphane complexes

**Fig. 6:**
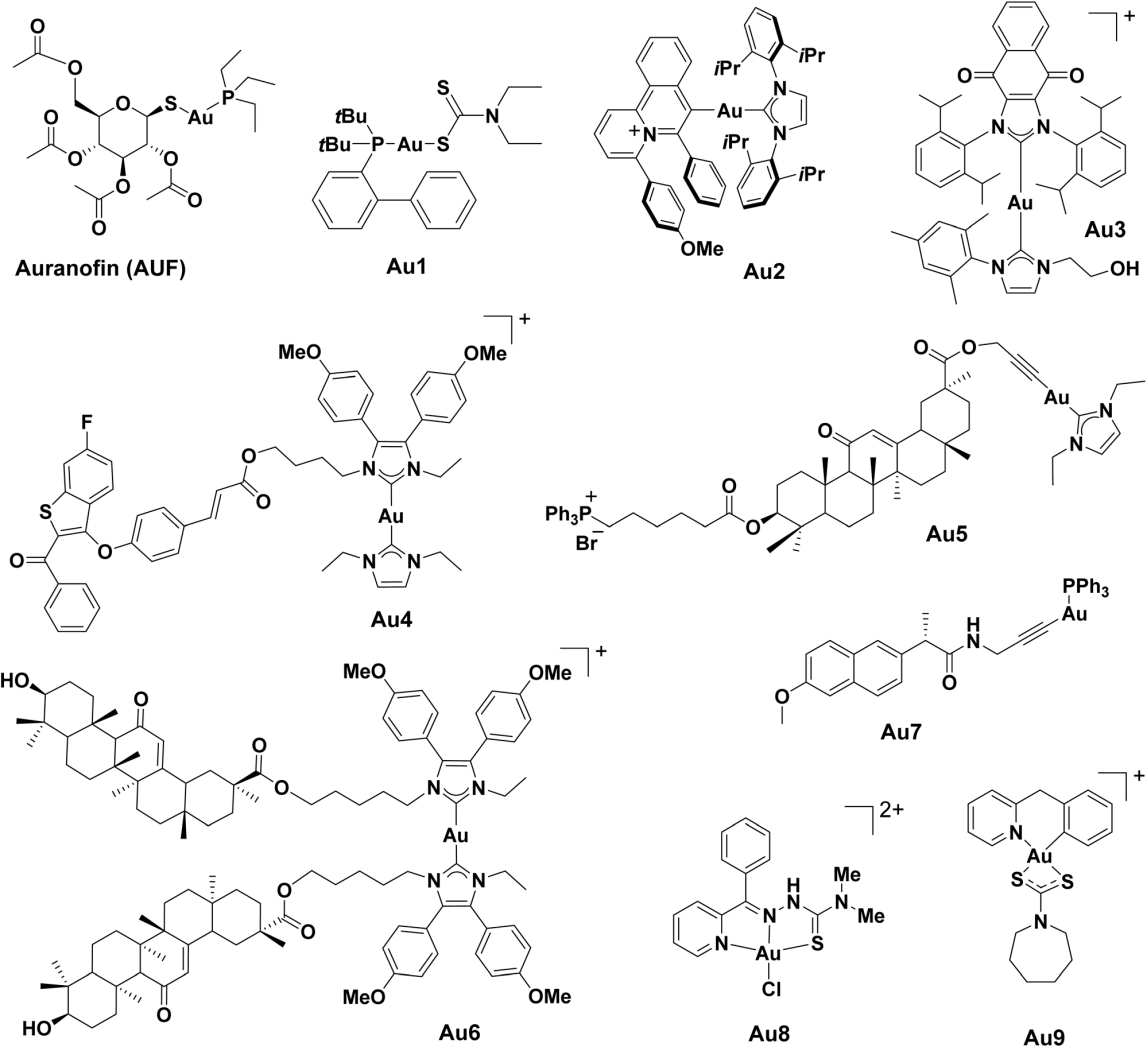
Molecular structures of reported Au-based ICD inducers. Counter anions are omitted for clarity.

Owing to their unique chemical properties, Au complexes could effectively inhibit thioreductase (TrxR), a Se-containing enzyme responsible for redox homeostasis, leading to intracellular ROS generation.^238^ Multiple Au complexes were reported to induce ICD using this approach.^75–77,82,182^ Isab and Ang *et al.* reported the first Au(i) complex, an Au(i)-phosphane dithiocarbamate complex (Au1, Fig. 6) that was able to induce a dose-dependent ecto-CRT exposure resulting from PERK-mediated eIF2α phosphorylation to initiate an immune response in ovarian cancer cells.^239^

Boullosa *et al.* reported that the FDA-approved auranofin (AUF) triggered ICD by inducing both apoptosis and ferroptosis in mutant p53 NSCLC cells *in vitro*.^240^ The treatment of AUF significantly induced DAMPs emission, and the co-culture of AUF-treated cancer cells with immature DCs led to their maturation. AUF further improved the innate immune response, as evidenced by the enhanced killing of cancer cells when they were co-cultured with natural killer cells. The group also reported that the combination of AUF and cold atmospheric plasma-treated PBS resulted in a synergistic ICD response in the glioblastoma cell culture.

#### 3.3.2 Organometallic Au(i) complexes

Patil *et al.* identified Au2 as a potential Au(i) ICD inducer.^241^ Following the optimization of cytotoxicity and ATP release, a library of 40 Au(i)-NHC complexes were generated by placing different benzo[*a*]quinolizinium (BQ) cores, ligands and counterions on Au. Among them, Au2 displayed the greatest potential in NSCLC A549 cells, demonstrating a dose-dependent increase in ICD biomarkers. Further evaluation of Au2 in human immune cells was conducted in co-culture with hPBMCs from healthy donors and phagocytosis assays with differentiated THP1 macrophages, and Au2-enhanced immunogenicity of A549 cells was observed.

A rationally designed redox-active Au(i)-NHC complex Au3 exhibited potent ICD induction efficacy *in vitro* and *in vivo*.^242^ The authors postulated that dual targeting of the cancer antioxidant network through TrxR inhibition by the redox-active Au(i)-NHC motif and redox cycling *via* the embedded naphthoquinone moiety could increase ROS generation and ER stress to promote ICD induction. In a vaccination model using mice inoculated with treated CT26 cells, low dose Au3 (10 μM) demonstrated a significantly higher percentage of tumour-free mice compared to high dose OXP (150 μM), even after extended periods of recovery post-challenge (42 days).

Moreover, several Au(i) complexes targeting the TrxR-ROS-ERS-ICD axis were reported by Liu and co-workers. For example, the *in vitro* ICD effects of Au(i)-NHC complex incorporating selective estrogen receptor degrader (SERD) moiety (G1T48) Au4 were studied in human MCF7 cells.^243^ The Au(i)-NHC moiety in Au4 was designed to inhibit TrxR activity, which consequently triggered ROS generation and ICD-associated DMAP emission, including CRT exposure, HMGB1 release, and ATP secretion. In addition, an NHC-Au(i) complex with liver-targeting scaffold 18β-glycyrrhetinic acid and mitochondria-directing triphenyl-phosphonium group (TPP^+^) Au5 was discovered to simultaneously induce both ICD and cGAS-STING pathways to trigger an immune response.^244^*In vivo* vaccination studies with Au5 produced a stable population of 50% tumour-free mice after 30 days. Another Au(i)-NHC with the same 18β-glycyrrhetinic acid ligand Au6 displayed a significant emission of DAMPs in Hepa1-6 cells after treatment.^245^ In particular, the Au6-treated cells saw no tumour growth for 30 days in an *in vivo* vaccination model. The treatment also increased the number of CD8^+^ T-cells and CD4^+^ T-cells by 3.9-fold and 5.6-fold, respectively.

Alkynyl ligands are widely used to stabilize Au(i) complexes owing to their strong electron donating abilities. Besides Au6, Liu and co-workers also designed a series of Au(i)-alkynyl complexes conjugated to nonsteroidal anti-inflammatory drugs (NSAID) with the aim of inhibiting TrxR activity and disrupting redox balance.^246^ Amongst these 7 Au(i)-NSAID complexes, naproxen-containing Au7 triggered oxidative stress and ICD-associated DAMPs emission in human A2780 cells and elicited a more effective immune response, inducing the downregulation of cyclooxygenase-2 (COX-2) and PD-L1, DC maturation and increased infiltration of CTLs.

#### 3.3.3 Au(iii) complexes

Au(iii) complexes possess different coordination geometries from Au(i) congeners and are usually kinetically less stable. Thus, they are typically stabilized with chelating ligands and “soft” binding partners. Recently, an Au(iii) 2-benzoylpyridine thiosemicarbazone complex Au8 was shown to induce ICD.^247^ Apart from ER stress and ROS generation, the complex demonstrated the ability to cause severe mitochondrial damage, resulting in apoptosis. ICD-associated DAMPs were detected in SKOV-3 cells in both *in vitro* and *in vivo* models. Although *in vivo* anti-tumour efficacy was studied, the absence of a vaccination model in the context of ICD assessment prevents the validation of its effectiveness as an ICD inducer.

Babak, Berger and Ang *et al.* utilized novel Au(iii)-thiocarbamate scaffolds to develop ICD inducers with superior efficacy and can reverse immunosuppressive TME. By applying a combinatorial coordination chemistry approach, a library of 35 cyclometalated Au(iii)-thiocarbamate complexes was constructed, and their ability to inflict ICD effects was assessed in a malignant pleural mesothelioma (MPM) cell model.^248^ A systematic structure–activity relationship study revealed that the cyclometalated scaffold and the overall lipophilicity of the complexes are crucial for the phagocytosis of immunologically “cold” MPM cells upon treatment. A *bona fide* Au(iii)-based inducer Au9 was successfully identified from the library, as evidenced by a robust antitumour immune response against MPM in immunocompetent mice. Protective antitumour immunity was observed for more than 6 months in these mice, demonstrating the viability of this Au(iii) scaffold as a discovery platform for ICD inducers.

### 3.4 Ru-based ICD inducers

#### 3.4.1 KP1339/IT-139/NKP1339/BOLD-100

**Fig. 7:**
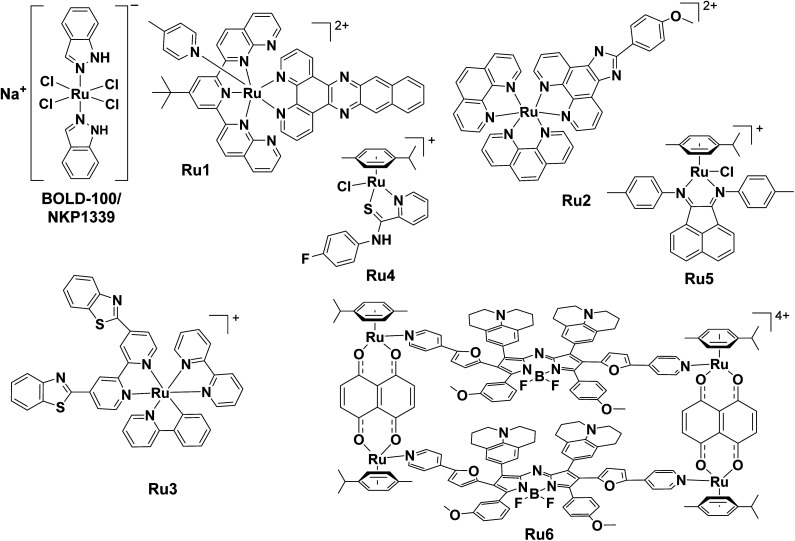
Molecular structures of reported Ru-based ICD inducers. Counter anions are omitted for clarity.

One of the earlier Ru-based ICD inducer discovered was sodium *trans*-[tetrachloridobis(1*H*-indazole)-ruthenate(iii)] (KP1339/IT-139/NKP1339/BOLD-100), a Ru(iii) drug candidate under clinical investigation (Fig. 7).^249^KP1339 is postulated to act *via* a prodrug mechanism through reduction to active Ru(ii) species after aquation.^250,251^ Its redox chemistry is believed to be important determinant of its anticancer activity.^252–254^KP1339 binds to multiple biomolecules including serum proteins (*e.g.* albumin and transferrin), the ER ribosomal proteins (*e.g.* RPL10, RPL24) and the transcription factor GTF2I as evidenced in target profiling experiments,^250,255,256^ as well as Bip (also known as GRP78).^257^ As an ICD inducer, interaction with RPL10 and RPL24 led to ribosomal disturbance, while binding to GRP78 caused ROS generation. This triggered ER stress was marked by elevated phosphorylation of eIF2α and PERK, which ultimately induced ICD. The ICD-inducing capacity of KP1339 was amplified upon loading into glutathione (GSH)-responsive nanocarrier, as evidenced by enhanced emission of DAMPs compared to KP1339 alone.^258^ The KP1339-loaded nanocarrier inhibited primary and distant tumour growth with low systemic toxicity and prevented pulmonary metastasis of breast cancer.

#### 3.4.2 Ru(ii)-polypyridyl complexes

Similar to Ir(iii)-poylpyridyl complexes, Ru(ii)-polypyridyl complexes also constitute a class of important photosensitizers for PDT owing to their tuneable photophysical and biological properties, which have been exploited for ICD induction. These Ru(ii) photosensitizers usually possess a tridentate polypyridyl ligand for near-infrared absorbance, a bidentate π-expanded *N*,*N*-ligand for sensitizing singlet oxygen and a monodentate ligand to fine-tune its overall properties. One such compound Ru1 demonstrated superior ICD-inducing capacities both *in vitro* and *in vivo* under NIR activation.^259–261^ The potent efficacy of Ru1 on tumour growth inhibition, enhancing mice survival, and antitumour immunity in the vaccination model suggested a potential for its clinical utility.

Another study described the ability of Ru(ii)-polypyridyl complex Ru2 in increasing ecto-CRT in mice although other ICD hallmarks were not measured.^262^ Intriguingly, the combination of Ru2 and natural killer (NK) cells led to the surprising finding that this combination treatment could foster NK cell infiltration, potentiate NK cell immunotherapy, and improve therapeutic efficacy against breast tumour *in vivo*. This study highlighted the immunoregulatory effects of Ru2 as a potential ICD inducer and provided an innovative angle for applying ICD inducers to augment immunotherapy.

Chao and co-workers designed 3 cyclometalated Ru(ii) complexes and found that Ru3 targeted mitochondria and nucleus, leading to oncosis accompanied by ICD induction.^153^ Its mechanism of action involved DNA damage causing activation of polyADP-ribose polymerase 1 (PARP1), associated ATP depletion and porimin activation, as well as concurrent mitochondria damage and ER stress. More importantly, macrophage M1 polarization was observed, indicating the activation of innate immune response on top of adaptive T-cell response. However, the limited solubility and bioavailability of Ru3 necessitated an encapsulation approach *in vivo*.

#### 3.4.3 Organometallic Ru(ii) complexes

Plecstatin-1 Ru4 is an organoruthenium anticancer drug candidate capable of inducing oxidative stress and exerting ICD in tumour spheroids^263^ even though it specifically targets a scaffold protein and cytolinker, plectin.^264,265^ Besides CRT, HSP70 and HSP 90 were also translocated to the cell surface upon Ru4 treatment *in vitro*. Another half-sandwich Ru(ii) complex with aryl-bis(imino) acenaphthene Ru5 was identified as a *bona fide* ICD inducer in the vaccination model.^266^ ICD emission hallmarks were observed in melanoma cells. Its mode of action included mitochondrial impairment and metabolic reprogramming, leading to ER stress. A self-assembled supramolecule Ru6 based on piano-stool Ru(ii)-arene scaffold was constructed and validated as an ICD inducer.^267^ Upon NIR irradiation, Ru6 enabled highly concentrated and precise ROS generation in deep-seated tumour and induced ICD with all biochemical markers detected. Further, an *in vivo* vaccination assay shows CD4^+^/CD8^+^ T-cell responses and downregulated immunosuppression with more than a 4-fold reduction of Foxp3^+^ T-cells.

### 3.5 Other metal-based ICD inducers

#### 3.5.1 Re-based ICD inducers

**Fig. 8:**
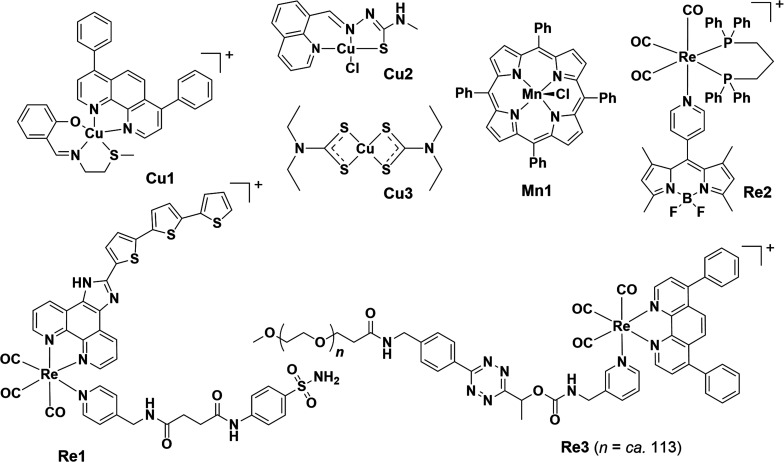
Molecular structures of reported Re, Cu and Mn-based ICD inducers. Counter anions are omitted for clarity.

Compared to Ir and Ru, Re complexes are less studied as photosensitizers, but their mechanism of action has been linked to ROS-associated ICD induction. Several interesting works have been reported on the design and modification of tricarbonyl Re(i) complexes to ICD inducers with impressive potency, theranostic function, or controlled activation modality (Fig. 8). The first Re-based ICD inducer, Re1, was purposefully designed with a CAIX anchor to destroy cancer cell membrane integrity *via* ROS generation upon photoirradiation at 425 nm.^268^Re1 exhibited remarkable photocytotoxicity in the nanomolar range against the MDA-MB-231 cell line under normoxia (20% O_2_) and hypoxia (1% O_2_) with negligible dark toxicity. ICD hallmarks were well-characterized *in vitro* by immunostaining and ATP detection assay. Cancer cells treated with Re1 underwent cell death *via* pyroptosis. Using a 4T1-bearing bilateral BALB/c mice model, the authors observed an increased percentage of matured (CD80^+^CD86^+^) DCs and elevated antigen-presenting capacity in Re1 suggested by a significant increase in TNF-α, IL-6 and IL-12p70 levels with a reduction in immunosuppressive cytokines IL-10 levels. Notably, the amount of tumour-filtrating CTLs and helper T-cells in tumour sites increased by 2–3 fold compared to that of the control (light only), suggesting the effective activation of an adaptive immune response. No systemic toxicity was observed in the experimental mice.

Tan *et al.* presented a theranostic Re(i) complex Re2 appended with 4,4-difluoroboradiazaindacene (BODIPY) moiety, which was used for viscosity measuring and imaging.^269^Re2 was preferentially localized in the ER, causing ER stress and, eventually, necrosis, and could be simultaneously used to monitor ER viscosity. Re2 was described as a Type II ICD inducer based on its ability to induce the 3 classical ICD biomarkers.

A Re(i) aminomethylpyridine complex Re3, modified with a cleavable tetrazine moiety which could be triggered by *trans*-cyclooct-4-enol (TCO-OH), underwent a click-to-release reaction, giving rise to a more cytotoxic Re(i) ICD inducer.^270^ In the presence of TCO-OH and light, the released Re(i) compound led to substantial ROS generation, lysosome rupture, autophagy inhibition, necrosis, and ICD induction with accompanying biomarkers. Notably, ATP secretion was observed even when autophagosome formation was blocked in the process, in contrast to a previous claim that ATP secretion in ICD relied on the autophagic process. Remarkable increases in the proportion of mature DCs (CD80^+^CD86^+^) and cytotoxic T-cells (CD3^+^CD8^+^), and the expression level of TNF-α were observed in a co-incubation of treated-MDA-MB-231 with hPBMCs, further proving its ability of immune activation.

A Re(i) photosensitizer Re4, constructed by coordinating [Re(CO)_3_]^+^ to g-C_3_N_4_ nanosheets (Re(i)–g-C_3_N_4_), was demonstrated as a Type II ICD inducer with ER-specific accumulation.^271^ Upon two-photon excitation, Re4 triggered robust ROS (˙O_2_^−^ and ˙OH)-driven ER stress, with different cell death modes, including apoptosis, ferroptosis, and pyroptosis, and most importantly, ICD-related DAMPs emission. Activation of antitumour immune responses together with inhibited growth of primary and secondary distant tumour in mice was observed.

#### 3.5.2 Cu-based ICD inducers

Cu is a crucial element involved in various physiological processes, such as cellular redox homeostasis and mitochondrial energy production. As one of the first-row transition metals able to initiate a Fenton-like reaction, Cu compounds are redox-active and contribute to ROS generation, thus perturbing redox homeostasis and causing cellular stress.^177^ Harnessing the redox activity of Cu to induce ICD and subsequent antitumour immune response has been documented, including Cu-based nanoparticles and small molecules. For example, to produce ROS precisely in the ER to result in potentiated ER stress, Suntharalingam *et al.* designed Cu complexes with different polypyridyl ligands with the aim of facilitating their distribution to the ER.^272^ Notably, a Cu(ii) complex containing a Schiff base ligand and a polypyridyl ligand Cu1 induced ICD in breast CSCs *via* ROS-driven ER stress, as evidenced by DAMPs emission, and promoted phagocytosis by macrophages. In another follow-up study, the same group encapsulated Cu1 into polymeric nanoparticles to enhance cellular uptake by CSCs and observed improved ICD efficacy.^273^

Another recent study also showcased the superior ICD inducing capacity of Cu(ii) complexes which can be attributed to their redox activities.^274^Cu2 depleted GSH forming monovalent Cu^+^ species that catalyzed ˙OH production *via* Fenton-like reaction. Replacing the center metal with Co, Pt or Pd resulted in the loss of cytotoxicity. Cu2-induced ICD was ferroptosis-dependent and enabled significant tumour growth prevention and effective antitumour immune response (increased CD8^+^ T-cell infiltration and decreased Foxp3^+^ T-cells) in vaccinated c57BL/6 mice challenged with colorectal cancer. Importantly, Cu2 exhibited cytotoxic specificity towards cancer cells only.

Another form of Cu-based ICD inducer is the combination of CuCl_2_ with an anti-alcoholism drug disulfiram (DSF/Cu).^169,275–279^ DSF was repurposed as an anticancer agent that readily formed active metabolite Cu-diethyldithiocarbamate complex Cu3, significantly enhancing the anti-tumour effects of DSF.^279–281^ DSF/Cu treatment was found to induce potent ICD in multiple cancers and ICD-based immune response against primary and rechallenged tumours.^169,275,277–279^ The ICD evoked by DSF/Cu was associated with cuproptosis, a newly characterized cell death characterized by the accumulation of Cu in mitochondria.^282,283^ Multiple lines of evidence showed that cuproptosis elicited ICD and enhanced the immunogenicity of dying tumour cells.^284–286^ In addition, DSF/Cu treatment drove the reprogramming and reversal of the immunosuppressive TME in humanized mice.^169,281^ It could be used in combination with αPD-L1 to enhance cancer immunotherapy^276^ and trigger radiation therapy-induced ICD when combined with radiation and chemotherapeutic agents.^169,277,278^ Some DSF/Cu combination therapies were investigated in clinical trials, such as NCT02671890 (Phase I, solid tumours and pancreatic cancer), NCT02715609 (Phase I/II, glioblastoma), and NCT02678975 (Phase II/III, glioblastoma).

#### 3.5.3 Mn-based ICD inducers

Mn(ii) can catalyze the decomposition of excess H_2_O_2_ in cells, yielding ˙OH *via* a Fenton-like reaction.^177^ However, investigation on ROS-ER stress-driven ICD inducers based on Mn remains rare. One recent study by Mao *et al.* found that Mn(iii) *meso*-tetraphenylporphyrin chloride Mn1 could induce ICD and autophagy while they were investigating its activity in regulating anion transport into cells.^287^ Upon treatment with Mn1 against HeLa cells, a 4-fold increase in intracellular Ca^2+^ concentration was observed. Classical ICD events, including relocation of CRT and ATP secretion, except for the liberation of HMGB1, were detected. Proteomics analysis revealed downregulation in natural anticoagulant proteins, suggesting the implication of an immune response. Despite a lack of mechanistic investigations and further validation, this study broadened the scope of metal-containing ICD inducers, being the first example of an Mn-based ICD inducer.

## 4. Current challenges and limitations

### 4.1 Understanding the molecular targets

Genome-wide CRISPR screening, RNA interference (RNAi), and multi-omic techniques (*e.g.* genomics, transcriptomics, proteomics and metabolomics) have been frequently used to investigate targets of metallodrugs.^227,264,288–294^ However, there are only few studies on target identification and validation for ICD complexes. A noteworthy example reported by Zou and co-workers disclosed binding immunoglobulin protein (*i.e.* Bip/GRP78), an abundant ER chaperone regulating protein homeostasis in the ER, as a potential therapeutic target in ICD induction by photoaffinity-based target profiling.^227^KP1339, OXP and a cyclometalated Ir(iii)-bisNHC complex Ir6 were found to interact with BiP, as evidenced by shifted *T*_m_ values in cellular thermal shift assay (CETSA). Apart from Bip, the functional proteins in the ER that could be promising targets for ICD inducers remains elusive.

Compounds with different metals will likely have different molecular targets and MOA, while ROS generation and ER stress are believed to be strongly associated with ICD induction.^75–77,82,182^ Multiple targets might be implicated in the continuous generation of ROS and ER stress provoked by metal complexes. The mystery of the molecular targets of respective metal-based ICD inducers and the relationship between their targets and ROS-driven ER stress-based ICD remains an intriguing topic in this field.

Meanwhile, because of the lack of understanding of the respective molecular targets in the induction pathway of ICD, the rational design of potent metallic ICD inducers based on structural optimization is challenging. Thus, the target profiling of respective metallo molecules is highly demanding and of great significance in accelerating the understanding of mechanistic mysteries in ICD. Notably, ICD inducers that simultaneously target multiple pathways to provoke ER stress can potentiate ICD effects. This suggests that the design of novel ICD inducers with multiple targets could be reasonable. Overall, unraveling the molecular targets of metal-based ICD inducers to aid in the rational design of more effective and potent ICD inducers is needed to bridge this research gap.

### 4.2 Comprehensive structure–activity relationship (SAR) studies

Most of the development approaches for metallo-ICD inducers rely on screening and only few studies have attempted a systematic investigation of the effects of structural changes on their ICD-inducing capacity (*e.g.*Pt-NHC and Au-NHC).^207,209,241^ The scarcity of SAR studies occurs for several reasons. First, identification of a potential ICD inducer *in vitro* requires successful detection of multiple DAMPs as well as effective activation of immune cells, but related experimental procedures are highly laborious and resource-intensive. Second, to ensure robust ICD induction, it is necessary to monitor DAMPs at different time points and several drug concentrations.^26,34,89^ Despite the establishment of transgenic screening platforms, screening for large compound libraries at different concentrations with different treatment durations is challenging in practice.^149^ Third, building up a reliable screening platform by genetic manipulation is not easy. Finally, owing to the complexity of immune regulatory pathways,^87^ overall immunogenicity derived from ICD and the degree of activated antitumour immunity *in vivo* varies depending on the immunostimulatory and immunoinhibitory DAMPs balance, which complicates analyses. For example, gemcitabine triggers immunostimulatory DAMPs emission but also concurrently promotes the release of prostaglandin E2 as an immunoinhibitory signal, thus failing to provoke an effective antitumour immune response *in vivo*.^295^ An integrated screening platform that enables high throughput and systematic evaluation of ICD candidates would accelerate the drug discovery process and facilitate more robust mechanistic investigations, shedding light on their unique SAR.

### 4.3 Better cell and animal test models for ICD

In the design and screening of ICD inducers, the type of cell line models used is also crucial. Certain cell lines overexpressing ATP-hydrolyzing enzymes (*i.e.* CD39 and CD73) are likely to exhibit compromised immunostimulatory activity of DAMPs.^122,296^ This is because CD39 and CD73 reduce extracellular ATP by converting ATP to adenosine monophosphate (AMP) and adenosine, respectively.^296,297^ The accumulation of immunosuppressive extracellular adenosine, together with the decrease in the level of chemotactic and immunostimulatory ATP, weakens antitumour immunity.^296^ As ATP release is a pivotal marker of ICD, the activity of these enzymes might compromise the outcomes of ICD induction. Depending on whether the focus is on enhancing immune activation or studying immune evasion, it is key to consider the type of cell line carefully when designing the model. Next, human and mouse cell lines may exhibit differing responses to ICD inducers. A comprehensive ICD study should include both human and murine cell lines to compare the immunogenic response or ICD markers and ensure translational relevance because the ultimate goal of ICD research is to develop effective treatments for human patients in clinical settings.

Beyond *in vitro* assays, ICD should be validated *in vivo* to assess the induction of an immune response.^162,163,295^ However, validation can only be conducted in animal models with murine cell lines, as human cancer cells are intrinsically incompatible with *in vivo* immunological studies. Although attempts are being made to allow proper evaluation of ICD in the human system, the current state-of-the-art approach to identifying *bona fide* ICD inducers is through vaccination assays with immunocompetent syngeneic mice. Numerous studies despite showing promising *in vitro* results often validate through subcutaneous tumour models,^219,223,234,243,244,246,247,262,298^ where the primary focus is on analyzing the tumour growth curve to infer immunogenicity. Although informative, this approach may not fully capture the complexity of the immune response induced by ICD inducers. We therefore encourage researchers to standardize their validation processes by utilizing vaccination assays in syngeneic models because this will improve the reliability of ICD studies and better guide the development of novel ICD inducers.

## 5. Conclusion and future perspectives

This review highlights the significant advancements that have been made in the investigation, development and understanding of metal complexes for ICD in the past 2 decades since the ICD phenomenon was originally discovered. Given the strong interest in this field of research in recent years, this trend is expected to continue. Moving forward, we anticipate that the focus of effort will be channeled towards rationalizing the design of ICD complexes and developing specific clinical applications.

One strategy to rationalize the design of ICD complexes is to consider the indispensable role of continuous ER stress in ICD initiation and, hence, to reinforce ER stress precisely *via* an ER targeting manner.^65,84–86,226,299–302^ In response to stressors, cancer cells initiate UPR to relieve stress and maintain ER homeostasis. Stressors can target and remain in the ER, invoke persistent ER stress and counteract stress relief. Multiple lines of evidence have shown the effectiveness of this method by modifying the ligand environment of metal compounds to ensure their accumulation in the ER^86,222,234,299,303^ or constructing ER targeting delivery systems to direct them into the ER.^65,85,226,301,302^

The effectiveness of ICD in potentiating antitumour immunity depends on tumour immunogenicity and the host immune system. Another strategy is to develop complexes that act on immune cells, such as DCs and T-cells, and facilitate their detection, recognition and interaction of cancer antigens, which can boost ICD-primed immune response. Such functional molecules, aptly called “ICD enhancers” by Kroemer and co-workers, include hexokinase-2 inhibitors (immunometabolic modifiers) and ligands of pattern recognition receptor 3 (TLR3).^304^ Overall, the augmentation of ICD effects can be achieved by amplifying ICD in cancer cells or enhancing the perception of ICD by immune cells, suggesting promising ways to augment ICD and improve ICD-based therapies.

ICD-primed antitumour immune responses were also observed in the context of non-apoptotic RCD, such as ferroptosis,^305–307^ necroptosis,^308,309^ cuproptosis,^285,286^ and pyroptosis.^303,310,311^ These new RCDs could provide the basis and inspiration for the design of new ICD complexes. For example, cancer cells undergoing glutathione peroxidase 4 inhibition-induced ferroptosis were found to be immunogenic and can elicit robust antitumour immunity *in vivo*.^305,306^ Necroptotic cancer cells generated by genetic manipulation emit DAMPs, promote DC maturation and cross-priming of CTLs, and trigger an antigen-specific antitumour immune response.^308^ Pyroptosis is viewed as an ICD modality characterized by DAMPs emission that can enhance antitumour immune responses. Despite the poor direct killing of cancer cells, the pyroptotic cell death activator (*i.e.* GSDMD agonist) is an effective booster when synergized with other cancer immunotherapy.^310^ Cuproptosis, a newly described cell death modality, occurs owing to the overload of Cu in mitochondria and has been linked to trigger ICD effects.^285,286^ Altogether, these studies highlight the significance of non-apoptotic RCD in ICD initiation and suggest a promising way to discover ICD inducers that trigger immunogenic non-apoptotic cell death. Although agents that lead to non-apoptotic RCD may not guarantee the discovery of *bona fide* ICD inducers, they are more likely to yield a successful ICD induction and effective antitumour immunity.^303,312–314^

Regarding clinical applications, a promising avenue for ICD inducers may lie in complementing existing T-cell-based immunotherapy specifically targeting cold tumours.^45,189,190,196,248,315–318^ Cold tumours, also known as immune-excluded tumours, are characterized generally by a low expression of PD-L1 and lack of tumour-infiltrating lymphocytes, and are thus poorly responsive to T-cell-based therapies, such as immune checkpoint inhibitors.^319^ This represents a significant gap in cancer immunotherapy. ICD is one of the therapeutic strategies that can promote T-cell priming in “cold” tumours. ICD inducers can change TME from a “cold” to “hot” immune status by enhancing the immunogenicity of the dead cells through the production of neoantigens, DAMPs and cytokines to recruit and activate APCs and effector T-cells (CD4^+^ and CD8^+^).^45,196,203,204,315,318,319^ The efficient release of antigen, along with antigen processing and presentation, can improve T-cell priming. Thus, this enables T-cells to be available in the tumour for future cancer cell elimination.

One example of ICD inducers described previously, PT-112, is effective against “cold” tumour.^203–205^ Its combination with ICIs such as CTLA4, PD1 and PD-L1 blockers has shown a synergistic effect and can induce a more potent immune response compared to either therapy alone *in vivo*. The study also shows that PT-112 favored the establishment of an immunostimulatory tumour microenvironment. Collectively, the combination of PT-112 with immune checkpoint inhibitors suggests a promising immunotherapy with improved clinical safety and efficacy for overcoming “cold” tumour. Besides PT-112, the cyclometalated Au(iii) complex Au9 has demonstrated the ability to boost the immune response against MPM cells, which can be classified as immunologically “cold tumours” owing to poor responses to the immune checkpoint blockade combination treatment. In a preclinical study involving vaccinated mice, Au9 extended the tumour-free survival period to 5–7 months. Although lacking clinical investigation, this example highlights the potential of ICD inducers in targeting cold tumours that are less receptive to immunotherapies and hence are limited to chemotherapy. Apart from PT-112 and Au9, a few metallic ICD inducers have been shown to upregulate the level of PD-L1 in cancer cell lines that are classified as “cold” tumour in recent years.^198,199,223,225,234,246^ The combination of these complexes and PD-1 displayed a synergistic effect *in vivo*, with the combination significantly upregulating the level of mature DCs, T-cell infiltration and cytokines while downregulating immunosuppressive inflammatory cytokines, signifying the transformation into “hot” tumour.

These studies underscore the potential of ICD inducers as promising agents for the treatment of cancers that display limited sensitivity to ICIs. Thus, enhancing the immunogenicity with ICD inducers while simultaneously reducing immunosuppression through ICIs offers a promising approach to convert the TME from an immunosuppressive “cold” to an immunostimulatory “hot” environment that can generate a robust immune response.

## Author contributions

Jiao Xia Zou – original draft, figures and table, data procuring, editing, citing, formatting; Meng Rui Chang – original draft, data procuring: Nikita A. Kuznetsov – writing; Jia Xuan Kee – writing, review & editing. Prof. Wee Han Ang and Prof. Maria V. Babak: writing, formatting, review & editing.

## Conflicts of interest

There are no conflicts to declare.

## Data Availability

No primary research results, software or code have been included and no new data were generated or analysed as part of this review.
